# Bis[2-(2-amino­ethyl­amino)­ethanol]copper(II) dinitrate

**DOI:** 10.1107/S1600536811030637

**Published:** 2011-08-06

**Authors:** Reza Azadbakht, Hadi Amiri Rudbari, Giuseppe Bruno

**Affiliations:** a Department of Chemistry, Payame Noor University, Hamedan, Iran; b Dipartimento di Chimica Inorganica, Vill. S. Agata, Salita Sperone 31, Università di Messina 98166 Messina, Italy

## Abstract

In the title compound, [Cu(C_4_H_12_N_2_O)_2_](NO_3_)_2_, the central Cu^II^ atom has a distorted octa­hedral coordination geometry and is surrounded by four N atoms and two O atoms from the two inversion-related 2-(2-amino­ethyl­amino)­ethanol ligands. In the crystal, mol­ecules are held together by inter­molecular O—H⋯O and N—H⋯O hydrogen bonds, leading to the formation of a three-dimensional network.

## Related literature

For crystal structures of related complexes, see: Qu *et al.* (2004 ▶); Uçar & Bulut (2005 ▶); Chastain & Dominick (1973 ▶). 
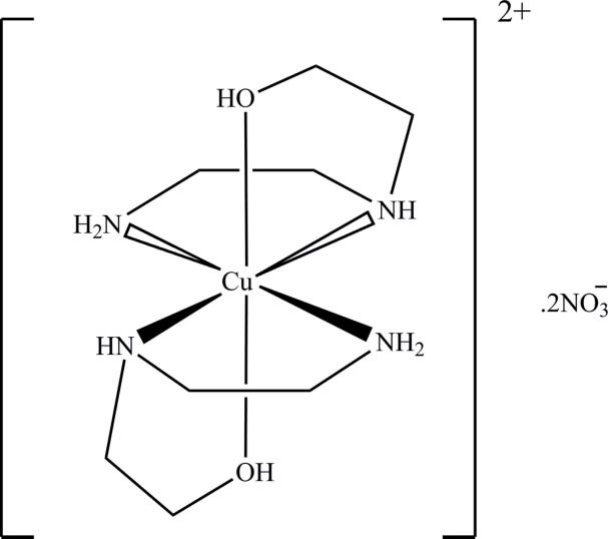


## Experimental

### 

#### Crystal data

[Cu(C_4_H_12_N_2_O)_2_](NO_3_)_2_
*M* *_r_* = 395.87
Tetragonal, 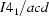

*a* = 14.6640 (1) Å
*c* = 29.8298 (7) Å
*V* = 6414.39 (16) Å^3^
*Z* = 16
Mo *K*α radiation
μ = 1.41 mm^−1^
*T* = 296 K
0.45 × 0.36 × 0.23 mm

#### Data collection

Bruker APEXII CCD diffractometer
Absorption correction: multi-scan (*SADABS*; Sheldrick, 2004 ▶) *T*_min_ = 0.532, *T*_max_ = 0.741
224935 measured reflections
4172 independent reflections
3346 reflections with *I* > 2σ(*I*)
*R*_int_ = 0.047

#### Refinement

*R*[*F* ^2^ > 2σ(*F* ^2^)] = 0.035
*wR*(*F* ^2^) = 0.121
*S* = 1.04
4172 reflections
109 parameters
H atoms treated by a mixture of independent and constrained refinement
Δρ_max_ = 0.67 e Å^−3^
Δρ_min_ = −0.72 e Å^−3^

### 

Data collection: *APEX2* (Bruker, 2009 ▶); cell refinement: *SAINT* (Bruker, 2009 ▶); data reduction: *SAINT*; program(s) used to solve structure: *SHELXS97* (Sheldrick, 2008 ▶); program(s) used to refine structure: *SHELXL97* (Sheldrick, 2008 ▶); molecular graphics: *SHELXTL* (Sheldrick, 2008 ▶); software used to prepare material for publication: *SHELXTL*.

## Supplementary Material

Crystal structure: contains datablock(s) I, global. DOI: 10.1107/S1600536811030637/qm2020sup1.cif

Structure factors: contains datablock(s) I. DOI: 10.1107/S1600536811030637/qm2020Isup2.hkl

Additional supplementary materials:  crystallographic information; 3D view; checkCIF report

## Figures and Tables

**Table 1:** Hydrogen-bond geometry (Å, °)

*D*—H⋯*A*	*D*—H	H⋯*A*	*D*⋯*A*	*D*—H⋯*A*
N2—H2*A*⋯O3^i^	0.90	2.14	2.9963 (17)	159
O1—H1⋯O4^ii^	0.93	2.28	3.1256 (16)	152
N2—H2*B*⋯O4^iii^	0.90	2.58	3.3356 (18)	141
N1—H2⋯O4^iv^	0.92 (2)	2.49 (2)	3.2449 (18)	139.8 (18)

Symmetry codes: (i) 
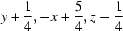
; (ii) 
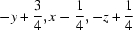
; (iii) 
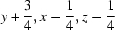
; (iv) 
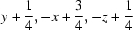
.

**Fig. 1.**
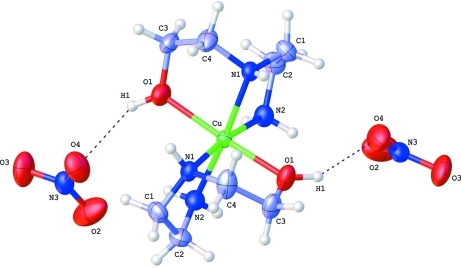
The structure of title compound, showing 50% probability displacement ellipsoids and the atom-numbering scheme.

**Fig. 2.**
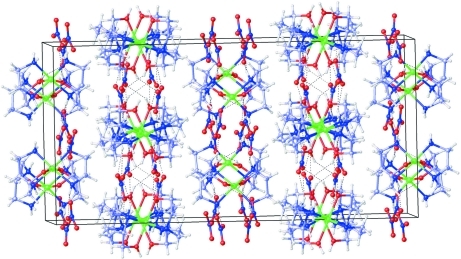
The crystal packing of title compound, viewed along the a axis. Hydrogen bonds are indicated by dashed lines.

## supplementary crystallographic information

### Comment

Metal alkanolamines complexes are among the most investigated compounds in coordination chemistry. As an extension of the work, we report here the crystal structure of the title compound, (I), a Cu^II^ complex incorporating the ligand *N*-(2-hydroxyethyl)ethylenediamine. The structure of bis [*N*-(2-hydroxyethyl)ethylenediamine] copper(II) nitrate consists of discrete [Cu(*L*)2]2+cations and nitrate anions. The closest distance between Cu and O of NO3 is 5.85 Å. The *ORTEP* diagram of the cation with the atom numbering scheme is shown in Fig. 1. The Ligand (*L*) coordinates in a tridentate manner *via* two nitrogen atoms and one oxygen atom, as shown in Fig. 1, providing a distorted octahedral arrangement about copper. The two O atoms coordinate to the Cu^II^ atom in *trans* positions, while the four N atoms occupy the equatorial positions. The three *trans* angles at the Cu^II^ atom are about 172° and the other angles subtended at the Cu^II^ atom are close to 90°, varying from 81.26 (15) to 95.15 (15)°. The two O atoms coordinate to the Cu^II^ atom in *trans* positions. The secondary-amine N-atoms and primary-amine N-atoms coordinate to the Cu^II^ atom in *trans* positions. In the crystal structure, the molecules are held together by intermolecular O—-H—O and N—-H—-O hydrogen bonds, leading to the formation of a three-dimensional network (Fig. 2 and Table 2).

### Experimental

Copper(II) nitrate dihydrate (0.5 mol) in 50 ml of methanol was slowly mixed with *N*-(2-hydroxyethyl)ethylenediamine (1 mol) in 50 ml of methanol. The reaction was refluxed for a further 2 h. The solution volume was then reduced to 10 ml by roto-evaporation. Vapour diffusion of ether into this solution afforded pink crystals.

### Refinement

The H-atoms were included in calculated positions and treated as riding atoms: O—H = 0.93 Å, C—-H=0.97 Å, N—H = 0.93and 0.90 Å for NH and NH2, respectively, with *U*_iso_(H) = k × *U*_eq_(C), where k = 1.5 for OH and CH3 H-atoms and k = 1.2 for all other H-atoms.

### Crystal data

**Table:**

[Cu(C_4_H_12_N_2_O)_2_](NO_3_)_2_	*D*_x_ = 1.640 Mg m^−^^3^
*M**_r_* = 395.87	Mo *K*α radiation, λ = 0.71073 Å
Tetragonal, *I*4_1_/*a**c**d*	Cell parameters from 9080 reflections
Hall symbol: -I 4bd 2c	θ = 3.1–36.4°
*a* = 14.6640 (1) Å	µ = 1.41 mm^−^^1^
*c* = 29.8298 (7) Å	*T* = 296 K
*V* = 6414.39 (16) Å^3^	Regular, pink
*Z* = 16	0.45 × 0.36 × 0.23 mm
*F*(000) = 3312	

### Data collection

**Table:**

Bruker APEXII CCD diffractometer	4172 independent reflections
Radiation source: fine-focus sealed tube	3346 reflections with *I* > 2σ(*I*)
graphite	*R*_int_ = 0.047
φ and ω scans	θ_max_ = 37.5°, θ_min_ = 2.4°
Absorption correction: multi-scan (*SADABS*; Sheldrick, 2004)	*h* = −24→24
*T*_min_ = 0.532, *T*_max_ = 0.741	*k* = −24→24
224935 measured reflections	*l* = −50→50

### Refinement

**Table:**

Refinement on *F*^2^	Primary atom site location: structure-invariant direct methods
Least-squares matrix: full	Secondary atom site location: difference Fourier map
*R*[*F*^2^ > 2σ(*F*^2^)] = 0.035	Hydrogen site location: inferred from neighbouring sites
*wR*(*F*^2^) = 0.121	H atoms treated by a mixture of independent and constrained refinement
*S* = 1.04	*w* = 1/[σ^2^(*F*_o_^2^) + (0.0796*P*)^2^ + 2.0215*P*] where *P* = (*F*_o_^2^ + 2*F*_c_^2^)/3
4172 reflections	(Δ/σ)_max_ = 0.001
109 parameters	Δρ_max_ = 0.67 e Å^−^^3^
0 restraints	Δρ_min_ = −0.72 e Å^−^^3^

### Special details

**Table:**

Geometry. All s.u.'s (except the s.u. in the dihedral angle between two l.s. planes) are estimated using the full covariance matrix. The cell s.u.'s are taken into account individually in the estimation of s.u.'s in distances, angles and torsion angles; correlations between s.u.'s in cell parameters are only used when they are defined by crystal symmetry. An approximate (isotropic) treatment of cell s.u.'s is used for estimating s.u.'s involving l.s. planes.
Refinement. Refinement of *F*^2^ against ALL reflections. The weighted *R*-factor *wR* and goodness of fit *S* are based on *F*^2^, conventional *R*-factors *R* are based on *F*, with *F* set to zero for negative *F*^2^. The threshold expression of *F*^2^ > 2σ(*F*^2^) is used only for calculating *R*-factors(gt) *etc*. and is not relevant to the choice of reflections for refinement. *R*-factors based on *F*^2^ are statistically about twice as large as those based on *F*, and *R*- factors based on ALL data will be even larger.

### Fractional atomic coordinates and isotropic or equivalent isotropic displacement parameters (Å^2^)

**Table:**

	*x*	*y*	*z*	*U*_iso_*/*U*_eq_	
Cu	0.7500	0.309239 (12)	0.0000	0.02305 (7)	
O1	0.61649 (7)	0.29396 (8)	−0.02849 (4)	0.0422 (2)	
H1	0.5640	0.3209	−0.0170	0.051*	
O2	0.77057 (12)	0.25081 (15)	0.19377 (6)	0.0770 (5)	
O3	0.71878 (11)	0.38235 (9)	0.21338 (6)	0.0619 (4)	
O4	0.62849 (9)	0.26916 (9)	0.20917 (4)	0.0566 (3)	
N1	0.71690 (7)	0.21768 (7)	0.05303 (3)	0.02816 (17)	
N2	0.79384 (7)	0.40760 (7)	−0.04633 (4)	0.03194 (19)	
H2A	0.7556	0.4556	−0.0460	0.038*	
H2B	0.8499	0.4275	−0.0388	0.038*	
N3	0.70700 (9)	0.29975 (8)	0.20544 (4)	0.0358 (2)	
C1	0.66926 (10)	0.27016 (10)	0.08834 (4)	0.0377 (3)	
H1A	0.6779	0.2401	0.1170	0.045*	
H1B	0.6044	0.2710	0.0819	0.045*	
C2	0.79595 (10)	0.36737 (10)	−0.09140 (5)	0.0388 (3)	
H2C	0.8356	0.4029	−0.1107	0.047*	
H2D	0.7352	0.3678	−0.1042	0.047*	
C3	0.61890 (9)	0.23541 (11)	−0.06844 (5)	0.0411 (3)	
H3A	0.6231	0.2731	−0.0951	0.049*	
H3B	0.5629	0.2004	−0.0702	0.049*	
C4	0.80093 (10)	0.17140 (11)	0.06673 (6)	0.0441 (3)	
H4A	0.8140	0.1225	0.0458	0.053*	
H4B	0.7921	0.1444	0.0961	0.053*	
H2	0.6816 (15)	0.1700 (17)	0.0429 (7)	0.054 (6)*	

### Atomic displacement parameters (Å^2^)

**Table:**

	*U*^11^	*U*^22^	*U*^33^	*U*^12^	*U*^13^	*U*^23^
Cu	0.01921 (9)	0.02340 (9)	0.02654 (10)	0.000	0.00019 (5)	0.000
O1	0.0266 (4)	0.0506 (5)	0.0494 (6)	0.0057 (4)	−0.0040 (4)	−0.0110 (5)
O2	0.0687 (9)	0.0947 (13)	0.0675 (9)	0.0433 (9)	−0.0148 (7)	−0.0199 (9)
O3	0.0572 (7)	0.0336 (5)	0.0948 (11)	−0.0136 (5)	−0.0136 (7)	0.0028 (6)
O4	0.0502 (6)	0.0520 (7)	0.0675 (7)	−0.0214 (5)	−0.0097 (5)	0.0097 (6)
N1	0.0238 (3)	0.0278 (4)	0.0328 (4)	−0.0029 (3)	0.0010 (3)	0.0025 (3)
N2	0.0318 (4)	0.0264 (4)	0.0376 (5)	−0.0005 (3)	0.0019 (4)	0.0050 (3)
N3	0.0378 (5)	0.0323 (5)	0.0374 (5)	−0.0004 (4)	−0.0086 (4)	0.0040 (4)
C1	0.0393 (6)	0.0418 (7)	0.0321 (5)	−0.0039 (5)	0.0098 (4)	0.0012 (5)
C2	0.0428 (6)	0.0409 (6)	0.0326 (5)	−0.0011 (5)	0.0032 (5)	0.0098 (5)
C3	0.0274 (5)	0.0523 (8)	0.0435 (6)	−0.0019 (5)	−0.0058 (4)	−0.0132 (6)
C4	0.0338 (6)	0.0386 (7)	0.0598 (9)	0.0033 (5)	0.0007 (6)	0.0213 (6)

### Geometric parameters (Å, °)

**Table:**

Cu—N2	2.0984 (10)	N2—H2A	0.9000
Cu—N2^i^	2.0984 (10)	N2—H2B	0.9000
Cu—N1^i^	2.1308 (10)	C1—C2^i^	1.517 (2)
Cu—N1	2.1308 (10)	C1—H1A	0.9700
Cu—O1	2.1460 (10)	C1—H1B	0.9700
Cu—O1^i^	2.1460 (10)	C2—C1^i^	1.517 (2)
O1—C3	1.4693 (17)	C2—H2C	0.9700
O1—H1	0.9300	C2—H2D	0.9700
O2—N3	1.2269 (19)	C3—C4^i^	1.505 (2)
O3—N3	1.2462 (16)	C3—H3A	0.9700
O4—N3	1.2406 (17)	C3—H3B	0.9700
N1—C4	1.4649 (17)	C4—C3^i^	1.505 (2)
N1—C1	1.4798 (17)	C4—H4A	0.9700
N1—H2	0.92 (2)	C4—H4B	0.9700
N2—C2	1.4684 (18)		
			
N2—Cu—N2^i^	93.16 (6)	H2A—N2—H2B	108.2
N2—Cu—N1^i^	82.79 (4)	O2—N3—O4	121.27 (17)
N2^i^—Cu—N1^i^	172.64 (4)	O2—N3—O3	121.15 (17)
N2—Cu—N1	172.64 (4)	O4—N3—O3	117.58 (15)
N2^i^—Cu—N1	82.79 (4)	N1—C1—C2^i^	111.89 (10)
N1^i^—Cu—N1	101.88 (6)	N1—C1—H1A	109.2
N2—Cu—O1	95.19 (5)	C2^i^—C1—H1A	109.2
N2^i^—Cu—O1	93.04 (4)	N1—C1—H1B	109.2
N1^i^—Cu—O1	81.26 (4)	C2^i^—C1—H1B	109.2
N1—Cu—O1	91.17 (4)	H1A—C1—H1B	107.9
N2—Cu—O1^i^	93.04 (4)	N2—C2—C1^i^	109.24 (10)
N2^i^—Cu—O1^i^	95.19 (5)	N2—C2—H2C	109.8
N1^i^—Cu—O1^i^	91.17 (4)	C1^i^—C2—H2C	109.8
N1—Cu—O1^i^	81.26 (4)	N2—C2—H2D	109.8
O1—Cu—O1^i^	168.02 (6)	C1^i^—C2—H2D	109.8
C3—O1—Cu	111.12 (7)	H2C—C2—H2D	108.3
C3—O1—H1	124.4	O1—C3—C4^i^	110.83 (11)
Cu—O1—H1	124.4	O1—C3—H3A	109.5
C4—N1—C1	116.07 (12)	C4^i^—C3—H3A	109.5
C4—N1—Cu	107.91 (8)	O1—C3—H3B	109.5
C1—N1—Cu	107.95 (8)	C4^i^—C3—H3B	109.5
C4—N1—H2	102.3 (14)	H3A—C3—H3B	108.1
C1—N1—H2	111.3 (14)	N1—C4—C3^i^	112.19 (11)
Cu—N1—H2	111.2 (14)	N1—C4—H4A	109.2
C2—N2—Cu	109.47 (8)	C3^i^—C4—H4A	109.2
C2—N2—H2A	109.8	N1—C4—H4B	109.2
Cu—N2—H2A	109.8	C3^i^—C4—H4B	109.2
C2—N2—H2B	109.8	H4A—C4—H4B	107.9
Cu—N2—H2B	109.8		
			
N2—Cu—O1—C3	79.30 (10)	O1^i^—Cu—N1—C1	105.41 (9)
N2^i^—Cu—O1—C3	172.76 (10)	N2^i^—Cu—N2—C2	−157.09 (10)
N1^i^—Cu—O1—C3	−2.56 (10)	N1^i^—Cu—N2—C2	16.74 (8)
N1—Cu—O1—C3	−104.40 (10)	O1—Cu—N2—C2	−63.74 (9)
O1^i^—Cu—O1—C3	−53.89 (10)	O1^i^—Cu—N2—C2	107.54 (9)
N2^i^—Cu—N1—C4	−117.16 (10)	C4—N1—C1—C2^i^	88.06 (14)
N1^i^—Cu—N1—C4	68.61 (10)	Cu—N1—C1—C2^i^	−33.15 (13)
O1—Cu—N1—C4	149.92 (10)	Cu—N2—C2—C1^i^	−38.94 (13)
O1^i^—Cu—N1—C4	−20.75 (10)	Cu—O1—C3—C4^i^	25.17 (15)
N2^i^—Cu—N1—C1	9.00 (8)	C1—N1—C4—C3^i^	−79.92 (15)
N1^i^—Cu—N1—C1	−165.23 (9)	Cu—N1—C4—C3^i^	41.32 (15)
O1—Cu—N1—C1	−83.92 (8)		

Symmetry codes: (i) −*x*+3/2, *y*, −*z*.

### Hydrogen-bond geometry (Å, °)

**Table:**

*D*—H···*A*	*D*—H	H···*A*	*D*···*A*	*D*—H···*A*
N2—H2A···O3^ii^	0.90	2.14	2.9963 (17)	159.
O1—H1···O4^iii^	0.93	2.28	3.1256 (16)	152.
N2—H2B···O4^iv^	0.90	2.58	3.3356 (18)	141.
N1—H2···O4^v^	0.92 (2)	2.49 (2)	3.2449 (18)	139.8 (18)

Symmetry codes: (ii) *y*+1/4, −*x*+5/4, *z*−1/4; (iii) −*y*+3/4, *x*−1/4, −*z*+1/4; (iv) *y*+3/4, *x*−1/4, *z*−1/4; (v) *y*+1/4, −*x*+3/4, −*z*+1/4.
